# Raman and XANES Spectroscopic Study of the Influence of Coordination Atomic and Molecular Environments in Biomimetic Composite Materials Integrated with Dental Tissue

**DOI:** 10.3390/nano11113099

**Published:** 2021-11-16

**Authors:** Dmitry Goloshchapov, Nikita Buylov, Anna Emelyanova, Ivan Ippolitov, Yuri Ippolitov, Vladimir Kashkarov, Yuri Khudyakov, Kirill Nikitkov, Pavel Seredin

**Affiliations:** 1 Solid State Physics and Nanostructures Department, Voronezh State University, University sq.1, 394018 Voronezh, Russia; goloshchapovdl@gmail.com (D.G.); buylov@phys.vsu.ru (N.B.); anna.liviero238@mail.ru (A.E.); vmkashkarov@gmail.com (V.K.); khudaykovyuri@rambler.ru (Y.K.); nikitkov.vsu@gmail.com (K.N.); 2 Department of Pediatric Dentistry with Orthodontia, Voronezh State Medical University, Studentcheskaya st. 11, 394006 Voronezh, Russia; stomat@vmail.ru (I.I.); dsvgma@mail.ru (Y.I.); 3 Scientific and Educational Center, Nanomaterials and Nanotechnologies, Ural Federal University, Mir av., 620002 Yekaterinburg, Russia

**Keywords:** biomimetic dental nanocomposites, mineralised tissue, enamel, dentine, Raman spectromicroscopy, XANES

## Abstract

In this work, for the first time, the influence of the coordination environment as well as Ca and P atomic states on biomimetic composites integrated with dental tissue was investigated. Bioinspired dental composites were synthesised based on nanocrystalline calcium carbonate-substituted hydroxyapatite $Ca_{4}^{I}Ca_{6}^{\mathrm{II}}{\left(PO_{4}\right)}_{6-x}{\left(CO_{3}\right)}_{x+y}{\left(OH\right)}_{2-y}$ (nano-cHAp) obtained from a biogenic source and a set of polar amino acids that modelled the organic matrix. Biomimetic composites, as well as natural dental tissue samples, were investigated using Raman spectromicroscopy and synchrotron X-ray absorption near edge structure (XANES) spectroscopy. Molecular structure and energy structure studies revealed several important features related to the different calcium atomic environments. It was shown that biomimetic composites created in order to reproduce the physicochemical properties of dental tissue provide good imitation of molecular and electron energetic properties, including the carbonate anion CO_3_^2−^ and the atomic Ca/P ratio in nanocrystals. The features of the molecular structure of biomimetic composites are inherited from the nano-cHAp (to a greater extent) and the amino acid cocktail used for their creation, and are caused by the ratio between the mineral and organic components, which is similar to the composition of natural enamel and dentine. In this case, violation of the nano-cHAp stoichiometry, which is the mineral basis of the natural and bioinspired composites, as well as the inclusion of different molecular groups in the nano-cHAp lattice, do not affect the coordination environment of phosphorus atoms. The differences observed in the molecular and electron energetic structures of the natural enamel and dentine and the imitation of their properties by biomimetic materials are caused by rearrangement in the local environment of the calcium atoms in the HAp crystal lattice. The surface of the nano-cHAp crystals in the natural enamel and dentine involved in the formation of bonds with the organic matrix is characterised by the coordination environment of the calcium atom, corresponding to its location in the Ca^I^ position—that is, bound through common oxygen atoms with PO_4_ tetrahedrons. At the same time, on the surface of nano-cHAp crystals in bioinspired dental materials, the calcium atom is characteristically located in the Ca^II^ position, bound to the hydroxyl OH group. The features detected in the atomic and molecular coordination environment in nano-cHAp play a fundamental role in recreating a biomimetic dental composite of the natural organomineral interaction in mineralised tissue and will help to find an optimal way to integrate the dental biocomposite with natural tissue.

## 1. Introduction

The study of the structure of biologically and artificially formed mineralised tissue has been developed extensively due to the relevance and importance of these data for medicine [1,2,3]. However, advances in restorative and regenerative dentistry, as well as the emergence of the biomimetic approach as a driving force for applied materials science, sometimes require the study of the properties of mineralised human hard tissue at the molecular and atomic level [4,5]. With respect to current medical technologies for dental hard tissue regeneration, it should be noted that despite the tremendous efforts that have been made to restore enamel using a variety of biomedical strategies and biocomposite materials, this task remains difficult [3,6]. The solution to enamel restoration involves understanding the molecular mechanisms of organisation and integration of artificial bioinspired materials based on nanocrystalline calcium carbonate-substituted hydroxyapatite (nano-cHAp) and natural tissue; finding the relationship between the inorganic mineral component and the organic matrix within which it was formed; and establishing the atomic ordering at the integration interface [3,5].

A lot of attempts have been made in order to reproduce an organomineral dental complex with the use of biomimetic principles. In the latter, biocomposites were created by the synthesis of nano-cHAp in the presence of different polymers and amino acid components [7,8,9,10,11,12,13]. This idea is based on the fundamental principles of organomineral interaction of the components in biocomposites and is utilized for the attainment of morphological uniformity and the homogeneous distribution of hydroxyapatite nanocrystals in the polymer and organic matrix. Amino acids were proven to be essential for the control of biomineralisation processes; however, every biosystem (bones, human teeth) requires its own set/ratio of different amino acids and the search for these sets/ratios represents the current goal of biomaterial science as well.

However, in spite of intensive investigations in this area of biomaterial science, approaches concerning the creation of materials with the functional characteristics of enamel and dentine still remain to be research-based, as most prospective current biomaterials are inferior to the native tissue. The main obstacle here is still the great existing difference in the functional properties of biocomposites relative to those that are inherent to hard natural tissues (low chemical tropism to the native tissue by its physical properties: mechanical strength, elasticity, optical and light emission characteristics, cytotoxicity [14,15,16,17,18]). All of these enumerated properties directly depend not only on the type of nanostructure defect and the morphology of calcium hydroxyapatite applied for their fabrication, but also on its hierarchical organization as well as on the type of interaction with the organic matrix in the biomimetic composite [19,20,21,22].

This complex problem is important for developing synthesis technologies and promoting nano-cHAp biomimetic materials in clinical practice.

The use of powerful material diagnostics that are sensitive to the molecular composition and local atomic environment is required to solve the problem of enamel restoration. In particular, incorporating the use of Raman microspectroscopy within the measuring scheme opens up broad analytical possibilities for investigating the mechanisms of molecular transformations in both single cells and biological tissues with high spatial resolution and positioning [23,24]. In contrast, changes in the local atomic environment that occur during the natural and artificial formation of mineralised hard tissues require specialised techniques for analysing biomaterials at the atomic level [25,26,27]. One such tool is X-ray absorption spectroscopy near the absorption edge (XANES). This analytical method allows for characterisation of the speciation (coordination and redox state) of the main elements of mineralised hard tissues (here, Ca and P in calcium phosphates) as well as associated carbonate ions and organic molecules. The combination of XANES and synchrotron radiation, a source of high-intensity polarised X-rays in a given energy range, facilitates studies at the angstrom level, which opens up new horizons for understanding biomineralisation processes [28,29,30].

Thus, our study aimed to investigate the influence of the atomic and molecular coordination environment and the state of the Ca and P atoms in biomimetic composites integrated with dental tissue.

## 2. Materials and Methods

### 2.1. Materials

The mineral basis of the bioinspired materials in our work was selected nanocrystalline cHAps, whose physicochemical properties are closest to the natural apatite of the dental matrix [31]. Samples of nano-cHAp were obtained using the wet chemistry method of titrating a concentrated solution of calcium hydroxide (Ca(OH)_2_) with 0.3 M orthophosphoric acid (H_3_PO_4_) solution. The raw calcium hydroxide was obtained by thermal annealing from a hen’s eggshells [31].

Since tooth enamel and dentine have different carbonate anion contents in the crystal structure [32,33], nano-cHAp samples (H_1_, H_2_, H_3_) with different percentages of CO_3_ substitution 0.3% < x < 2% were synthesised to determine the molecular and structural features.

The percentage of CO_3_ was monitored using ionometric techniques: by titrating the solution to the calculated value. In contrast, nano-HAP samples were obtained by the wet chemical route—СО_3_ inclusion into the crystalline lattice of HAP—and this process is determined by the type and concentration of reactants at the stage of synthesis as well as by titration rate [34,35,36]. In our work for the same titration rate of 0.025 mL/s for calcium hydroxide solution (Ca(OH)_2_) with 0.3М solution of orthophosphoric acid (H_3_PO_4_), the amount of H_3_PO_4_ varied in order to obtain a specified ratio of Са/P in HAp. Under a decrease in H_3_PO_4_ amount, an active inclusion of СО_3_ groups into the crystalline lattice of hydroxyapatite takes place. They substitute РО_4_ groups (B type of substitution) depending on the final Са/P ratio. The control for Са/P ratio is realized with the use of the ionometry technique, with the titration of samples up to the specified pH value and its stabilization for the period of 4 h (pH meter ionomer IPL 111-1, accuracy of 0.01). A continuous titration was performed due to the nature of Са(ОН)_2_, which was obtained in our work by mixing of thermally annealed hen’s eggs shell (950 °C) and distilled water [31].

Stoichiometric calcium hydroxyapatite (Hs) was obtained by hydrothermal techniques [37], with prolonged calcification and subsequent annealing to avoid the incorporation of CO_3_ into the hydroxyapatite (HAp) structure.

### 2.2. Creation of Biocomposites

Bioinspired composites were obtained using nano-cHAp with a percentage of CO_3_ of ~1.9%. The elementary polar amino acids of the dental amino acid matrix, found in enamel tubules, and hyaluronic acid, which has been shown to mineralise calcium hydroxyapatite when incorporated, were used to replicate the amino acid matrix of enamel and dentine. The amino acid cocktail (solution) consisted of L-arginine hydrochloride (C_6_H_15_ClN_4_O_2_), L-histidine, and L-lysine hydrochloride (C_6_H_15_ClN_2_O_2_) in a 12/1/3 ratio, with the addition of hyaluronic acid equal to ~2% of the total mass of the amino acid cocktail. All chemical components were purchased from Sigma-Aldrich (St. Louis, MO, USA). To obtain the amino acid solution, raw components were dissolved in ultra-pure water (provided by a Millipore Milli-Q gradient ultrapure water system) and then, subjected to ultrasound stirring (Q55 Sonica 55Вт) with an amplitude of 50% for 5 min.

The amino acid cocktail was mixed with nano-cHAp in an aqueous medium using ultrasound (55 W) for 5 min before introducing it into the amino acid solution. To reproduce the properties of enamel, a composite (C_E_) was prepared with an organic/mineral ratio of 5/95, whereas a bioinspired composite (C_D_) reproducing the properties of dentine had an organic/mineral ratio of 25/75. The organic/mineral ratio was specified at the stage of biocomposite synthesis as to the weight relation of the amino acid cocktail (organic part) to nano-cHAp (inorganic part).

### 2.3. Preparation of Natural Enamel and Dentine Samples

Samples of teeth extracted from patients for orthodontic indications were obtained from donors aged 22–28 years (5 males and 5 females). All participants taking part in the study were systemically healthy and had no unhealthy habits, as evidenced by their medical records. The samples of teeth had no carious areas or erosions; they were mechanically cleaned with a stiff brush after extraction and were stored in sodium azide solution at 4 °C until the experiments were carried out.

All the patients whose data were used within the created survey had signed institutional consent (protocol number: Pr13394, 16 December 2019) for their participation in the research. All the individuals who participated in the survey signed a written consent.

All of the experiments with human dental tissue and data collections were performed in accordance with relevant guidelines and regulations, including that all human participants provided informed consent for data collection and handling following the Helsinki Declaration. The study was approved by the Ethics Committee of Voronezh State University (Permission no. 001.017-2019, 21 December 2019).

### 2.4. Integration of Natural Dental Tissue and Biomimetic Composites (Treatment)

A cylindrical cavity was formed in the tooth by preparing the enamel layer down to the dentine using a micromotor dental drill. The rotational speed of the tungsten–vanadium bur was 4000 rpm. The speed was reduced when the dentine was reached. The cavity formed in the tooth was treated according to the technique described in our previous work [5]. Then, the C_D_ composite was applied to the dentine with universal adhesive, covering the entire area up to the visible edge of the dentin–enamel border and pre-cured with photopolymerisation. The C_E_ composite was then applied to cover the rest of the cavity in the enamel with universal adhesive. This was followed by photopolymerisation with UV light for 30 s.

**Figure 1:**
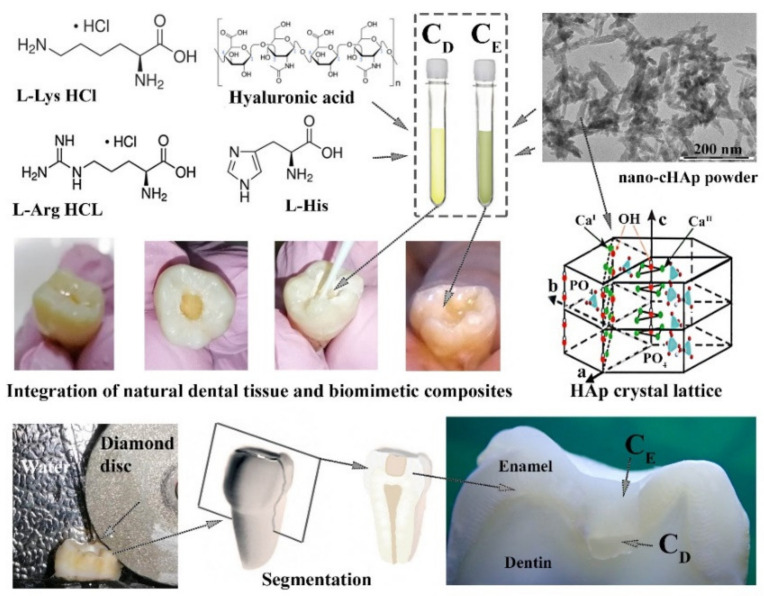
The schematic process of integration of natural dental tissue and biomimetic composites and segmentation of samples for spectroscopic studies. C_E_—biomimetic composite that imitates the properties of tooth enamel; C_D_—biomimetic composite that imitates the properties of tooth dentine.

The process of integration of natural dental tissue and biomimetic composites is schematically shown in Figure 1.

### 2.5. Segmentation of Samples

For spectroscopic studies of composites integrated with dental tissue, flat parallel slices containing natural tissue and the integrated biocomposite were cut from the treated samples (see Figure 1). A water-cooled diamond disc was used for this purpose. Subsequent sanding of the slices was performed according to our previously published technique [38].

### 2.6. Experimental Set-Up and Parameters

The molecular environment in the biomimetically mineralised composite was studied by Raman microspectroscopy. All spectra were obtained in the range of 100–1200 cm^−1^ using a confocal Raman microscope RamMix 532 (InSpectr, Moscow, Russia) with a spectral resolution of 1 cm^−1^. Excitation was performed using a 532 nm wavelength laser with ~30 mW of radiation power at the sample. Spatial resolution was 1μm.

X-ray absorption near edge structure (XANES) was carried out using equipment (Mark V, SRC, WI, USA) at the Synchrotron Radiation Center of the University of Wisconsin-Madison (Madison, WI, USA), in the total electron yield (TEY) mode. The vacuum inside the working chamber was of 10^−11^ Torr; instrumental broadening was of 0.05 eV and the depth of analysis was ~5 nm.

### 2.7. Data Collection and Spectral Processing

Raman and XANES spectral data processing, baseline correction, averaging, maximum positioning, integral area values and decomposition into components were performed using Origin 8 software (OriginLab Corporation, Northampton, MA, USA). Statistics was described using SigmaPlot 13.0 software (Systat Software, Inc., USA).

### 2.8. Description of the Samples Created and Investigated

Table 1 shows specifications of the samples of the synthesised biocomposites obtained by our research group, natural tooth tissue (enamel and dentine) and reference samples of natural tooth tissue (enamel and dentine) studied, as well as the standards of natural enamel and dentine of human teeth, according to literature data.

We prepared five (*n* = 5) specimens for investigations for each sample type.

It should be noted that the design of our research did not suppose a statistical analysis. Preliminary consideration of the obtained experimental spectral data demonstrated that the Raman spectra of the samples of one and the same type involve absolutely the same set of vibrational modes associated with certain molecular bonds, while XANES spectra are characterized by the same shape (profile) and a characteristic set of spectral features. Moreover, the spectra pertaining to a certain sample (type of specimens) can differ insignificantly by their intensity. This can be due to the individual distinctions of the persons who provided their biological tissues (enamel, dentin), as well as to the presence of the local micro-inhomogeneities on the surface of the samples. Taking all of these facts into account, we present the spectra averaged over a sample (type of specimens), while the following analysis is based on these spectral data.

## 3. Results

### 3.1. Raman Spectroscopy

**Figure 2:**
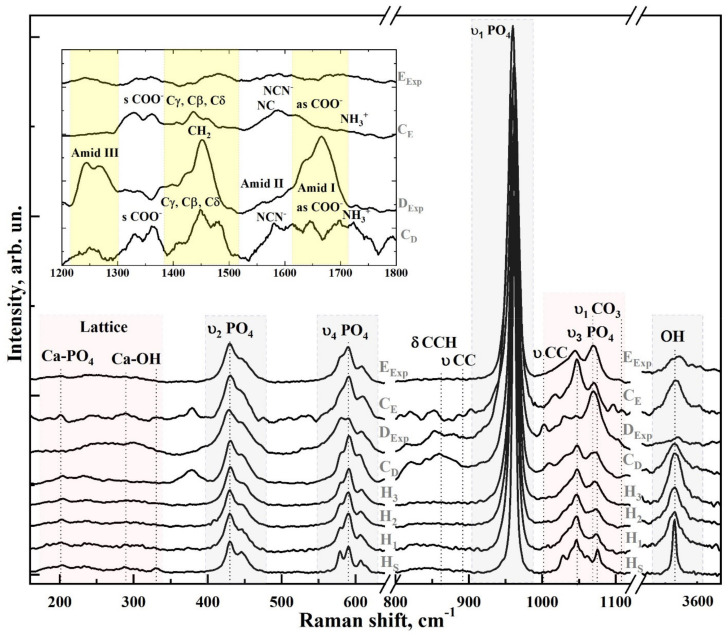
Typical Raman spectra of the investigated samples in the range of 120–1200 cm^−1^. The inset shows the range 1200–1800 cm^−1^ for the spectra of natural tissues and simulated bioinspired composites. Spectral features of six ranges are highlighted.

Figure 2 shows the Raman spectra of the reference samples, biomimetic composites and natural mineralised tissue (enamel and dentine). Raman spectra are given in the ranges of 100–1150 cm^−1^ and 3530–3630 cm^−1^, where the vibrations associated mainly with the mineral component of the samples are located. All of the spectra are normalised to the most intense band in the spectrum observed in the range 940–980 cm^−1^. In the inset of Figure 2, the spectra of natural hard tissues and the model composites in the range 1200–1800 cm^−1^ are presented, where vibrations associated with the organic component are present.

The frequencies of the main modes in the Raman spectra of the studied samples, as well as their relation to the vibrations of molecular groups and ions of the mineral and organic components of both the synthesised biomimetic and the natural composites, are presented in Table 2. The comparison was made based on data taken from the literature.

Analysis of the results shows that the main and most intense modes in the spectra of the natural tissues and the synthesised biocomposites can be attributed to the characteristic vibrations of cHAp (see Table 2), which is the basis of the mineral component of both the natural tissues and the biomimetic composites [32,40,43,46,47,48].

**Figure 3:**
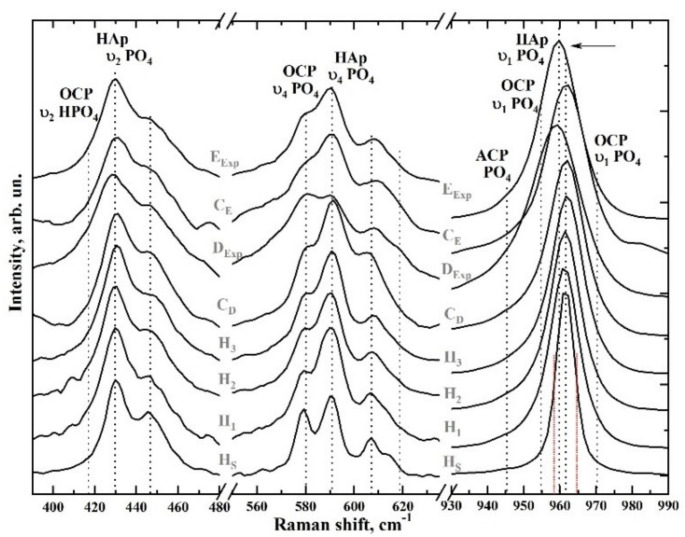
Raman spectra of the samples in the 380–480 cm^−1^ region (**left**), 550–630 cm^−1^ (**centre**), 930–990 cm^−1^ (**right**). In the right region (930–990 cm^−1^), the symmetrical valence vibration υ_1_ of PO_4_^2−^ is located. The centre and left part of the figure shows the spectral regions where the υ_2_ and υ_4_ PO_4_ bending modes are located.

**Figure 4:**
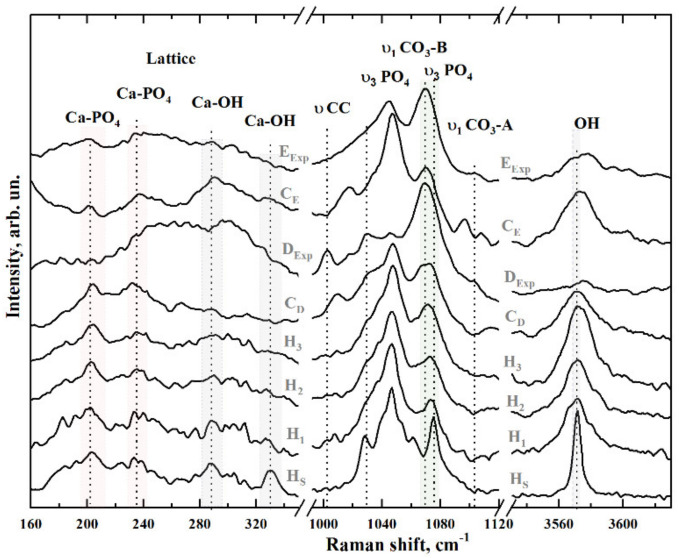
Raman spectra of samples in the regions 160–330 cm^−1^ (**left**), 1000–1120 cm^−1^ (**centre**) and 3540–3620 cm^−1^ (**right**). The region of 160–330 cm^−1^ corresponds to the lattice vibrational modes and modes of isolated ions of the mineral component in biogenic and synthetic composites are located. The vibrations of the OH group ions appear in the 3540–3620 cm^−1^ region. PO_4_ υ_3_ stretching modes are observed in the range 1000–1120 cm^−1^ (centre region).

More detailed spectral features within six ranges highlighted in Figure 2 are shown in Figure 3 and Figure 4. Figure 3 (right) shows the region 930–990 cm^−1^, where the most intense peak in the Raman spectra is located, ascribed to the symmetrical valence vibration υ_1_ of PO_4_^2−^ [40,43,46,47,48,49]. Figure 3 (centre and left) shows the spectral regions where the υ_2_ and υ_4_ PO_4_ bending modes are located. The first modes appear as two distinguishable peaks at 430 and 448 cm^−1^ and the second as four well-defined peaks at 580, 590, 608 and 619 cm^−1^, whose intensity depends on the sample type. In addition, a shoulder-like feature is observed in the Raman spectrum of dentine around 415 cm^−1^. This mode may be associated with the presence of small amounts of octacalcium phosphate (OCP) in the dentine tissue [43,44,45].

The position and full width at half-height (FWHM) of the vibrational mode of the υ_1_ PO_4_^2−^ oscillations differ from the spectra of the bioinspired materials (C_E_ and C_D_) and the natural tissues (E_Exp_ and D_Exp_) (Figure 3, right). For stoichiometric HAp (H_S_), it is centred around 961.5 cm^−1^; for synthesised nano-cHAp standards, the position of the main maximum is localised around 961.3, 961.7 and 962.1 cm^−1^ (samples H_1_, H_2_ and H_3_, respectively) and for bioinspired composites, around 961.8 cm^−1^. However, in the spectrum of natural enamel, this peak is shifted to 959.7 cm^−1^, and for natural dentine, an even greater shift to the 959 cm^−1^ region is observed, possibly due to the presence of a maximum in the 945 cm^−1^ region, which can be attributed to octacalcium phosphate and amorphous calcium phosphate (ACP) [43,44,45,48,51,52].

In Figure 4, there are three peaks correlated to the PO_4_ υ_3_ stretching mode (at 1028 cm^−1^, 1048 cm^−1^ and 1075 cm^−1^) in the range of 1000–1120 cm^−1^. These peaks have the lowest width in the spectrum of stoichiometric HAp (sample H_S_). It is known [32,33,40,46,53,54] that the increased content of carbonate groups in the hydroxyapatite structure causes the broadening of all bands in its Raman spectrum, resulting in the appearance of a wide band at around 1070 cm^−1^ with poorly resolved shoulders. This phenomenon is represented in the spectra of the reference samples of nano-cHAp, natural tissues and biocomposites (Figure 4). The described features in the spectra of enamel and dentine (E_Exp_, D_Exp_) are also present in the spectra of biomimetic samples (C_E_ and C_D_).

A peculiarity in the dentine spectrum around 1005 cm^−1^ may be ascribed to the νCC of the phenyl ring and the HPO_4_ group of both OCP and ACP, which agrees with previously published data [43,51,56]. The mode at 1018 cm^−1^ is also present in the spectrum of natural dentine and is typical for apatite containing divalent ions and vacancies instead of monovalent ions [39]. The broadening of the maximum at about 1070–1075 cm^−1^ in the spectrum of bioinspired materials is due to the overlap of two vibrational modes: the PO_4_ υ_3_ stretching mode and the υ_1_ mode of the carbonate anion СO_3_^2−^ included in the position of the PO_4_ group (B-type substitution), which is typical for biogenic materials [32,33,40,46,53,54]. A low-intensity oscillation in the region of 1103.4 cm^−1^ is also found in the spectra of samples of natural enamel and dentine of humans. This peak is attributed to υ_1_ of A-type СO_3_^2−^, when a carbonate anion appears in the OH position of the apatite group [32,43,46]. For biomimetic composites (C_E_ and C_D_), this oscillation is not observed.

The vibrations of the OH group ions appear in the Raman spectra as a mode around ~3570 cm^−1^ (see Figure 4). The most intense and narrowest peak is characteristic of the stoichiometric H_S_ hydroxyapatite sample. In contrast, for the other samples, this mode has a much wider width, and its intensity varies depending on the type of sample (reference nano-cHAp, mineralised natural tissues, biomimetic composites).

The region of 160–330 cm^−1^ (see Figure 4, left), in which both lattice vibrational modes and modes of isolated ions of the mineral component of biogenic and synthetic composites [39,41,42] are located, is of great interest in Raman spectra. This range is of general interest for understanding the substitution mechanisms in the crystalline structure of apatite [57], as well as for the evaluation of changes taking place in the coordination environment of the calcium atoms [41,57]. Thus, the modes located in the region of 150–250 cm^−1^ can be ascribed to Ca-PO_4_ bound vibrations of the HAp lattice and, as shown in Figure 4 (left), are most pronounced in the spectrum of the stoichiometric sample (H_S_). It is also known that the Ca_II_-OH bond in the HAp lattice manifests in Raman scattering spectra as vibrations around ~329, 305 and 270 cm^−1^ [42,57]. Analysis of the spectral data in the region of 160–330 cm^−1^ shows that for the stoichiometric sample of hydroxyapatite (H_S_), the spectral features related to Ca-PO_4_ and Ca_II_–OH have the same intensity. However, the maximum intensity in the range of 325 cm^−1^ for the synthesised nano-cHAp reference samples (H_1_, H_2_, H_3_) was lower than that for H_S_ and decreased with an increasing percentage of CO_3_ in the lattice (Table 2). The spectra of the samples do not show a significant difference in intensity and position of the Ca-PO_4_ vibrational modes in the range 200–237 cm^−1^, except for natural dentine (D_Exp_), which, in this area, is influenced by the non-apatite environment and the presence of ACP in the composition. At the same time, the spectral features of the Ca-PO_4_ modes in the spectra of the bioinspired composites are inherited from the reference nano-cHAp material used to create them. Moreover, for the enamel sample (E_Exp_), a broadening of the bands in the 200–237 cm^−1^ region and the absence of bands near 325 cm^−1^ can be noted. In the region of 3570 cm^−1^, a decrease in the intensity of the structurally bound OH group and appearance of low-intensity vibrations from carbonate anion υ_1_ СO_3_ in the position of the OH group is observed in the region of 1105 cm^−1^.

### 3.2. XANES Spectroscopy

**Figure 5:**
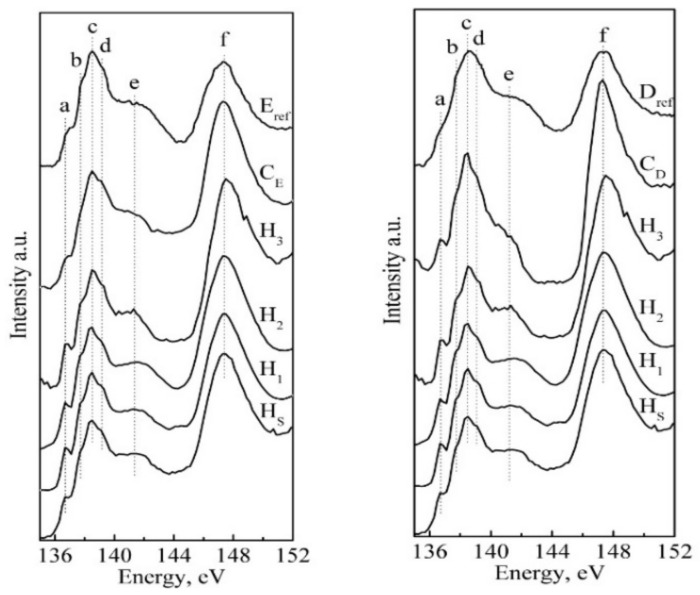
Phosphorus L-edge XANES spectra of reference samples, biomimetic composites and natural mineralised tissue (enamel and dentine). Spectra of reference enamel, a synthesised biomimetic composite that imitates the properties of tooth enamel and stoichiometric and carbonate-substituted hydroxyapatite are in the left part of the figure. Spectra of reference dentine, a synthesised biomimetic composite that imitates the properties of tooth dentine, stoichiometric and carbonate-substituted hydroxyapatite hydroxyapatites are in the right part.

Phosphorus L-edge spectroscopy is sensitive to the local chemical environment and oxidation of the absorbing atom [42,58]. Figure 5 shows the X-ray absorption spectra near the phosphorus L-edge of the nano-cHAp reference samples and biomimetic composites. In addition, Figure 5 shows the reference phosphorus L-edge for natural enamel and dentine samples from Srot et al. [25]. The spectra were normalised to the L3 edge. The features of the XANES spectra of the biomimetic composites reflect the shapes of the reference spectra (natural enamel and dentine) from [25]. The profile of the spectra of all samples is composed of the features indicated in the figures by the letters “a”, “b”, “c”, “d”, “e” and “f”; the positions of these features are shown in Table 3.

The positions of features “a” and “b”, which appear in the low-energy region of the spectrum, correspond to values 136.7 eV and 137.7 eV, and they are related to electron transitions from the *2p_3/2_* and *2p_1/2_* core levels to unoccupied *3s* states and are usually denoted as L_3_ and L_2_-edge [25,58,59]. Peaks “c” and “d” represent the spin–orbit splitting components of the dipole-forbidden transition to the *3p* states [60]. The broad feature “e” is a common characteristic of calcium phosphates, and is due to the transitions from P *2p* to unfilled Ca *3d* orbitals [25,59]. The “f” peak is interpreted as a form resonance and occurs when the absorbing phosphorus atom is coordinated with three or more electronegative atoms [61,62]; it is always located in the same energy position for all phosphates, whether they are crystalline or amorphous ones.

It should be noted that the spectra of the biomimetic composite, C_D_, and the reference dentine, D_Ref_, from Srot et al. [25], have several differences, which are possibly due to the crystal structure of HAp in the biocomposite. In addition, dentine contains crystalline HAp with a grain size of less than 10 nm, as well as ACP, which also contributes to the resulting spectrum.

**Figure 6:**
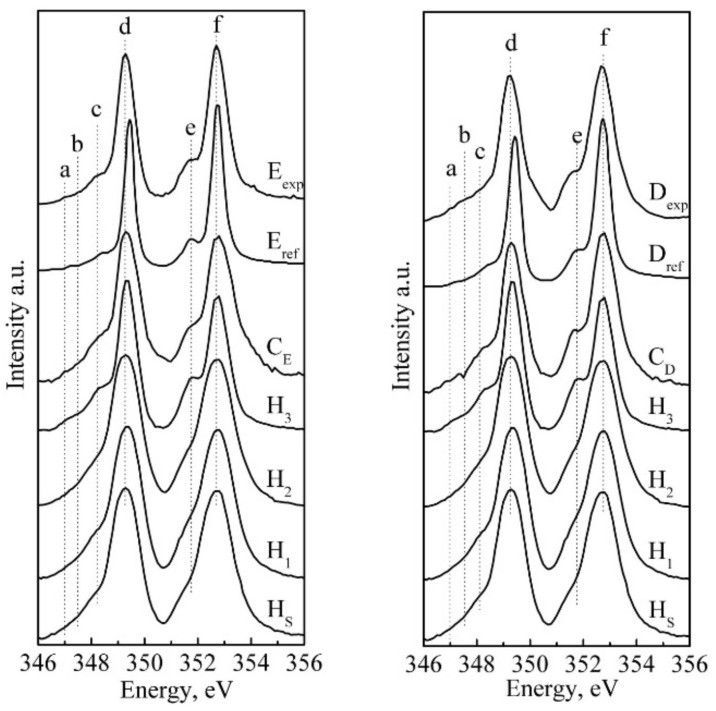
Ca L-edge XANES spectra of reference samples, biomimetic composites and natural mineralised tissue (enamel and dentine). Spectra of natural enamel, reference enamel, a synthesised biomimetic composite that imitates the properties of tooth enamel and stoichiometric and carbonate-substituted hydroxyapatite hydroxyapatites are in the left part of the figure. Spectra of natural dentine, reference dentine, a synthesised biomimetic composite that imitates the properties of tooth dentine, stoichiometric and carbonate-substituted hydroxyapatite hydroxyapatites are in the right part.

To understand the local Ca environment, calcium L-edge XANES spectra of reference samples, biomimetic composites and natural mineralised tissue (enamel and dentine) were obtained. Figure 6 also shows the reference calcium L-edge for natural enamel and dentine samples from Srot et al. [25]. The calcium L-edge spectra were normalised to the L_3_ edge. The shapes of all the experimentally obtained curves are comparable to the reference curves, and the main and high-intensity maxima “d” and “f” have similar positions, 349.3 eV and 352.7 eV, with slight variations (see Table 3) and correspond to the L_3_ and L_2_ calcium edges, respectively. However, the main difference in the spectra of the samples is the shape of the “c” and “e” pre-peaks (see Figure 6). In [25,63,64,65], it is specified that a less structured shape of features “c” and “e” is connected with low symmetry and indicates the amorphous structure of the sample. It is also noted in [63] that the ratio of the “e” pre-peak to the “f” peak is a good indicator of the environment closest to the absorbing Ca atom surrounded by oxygen atoms. The higher the “e”/“f” ratio is, the more ordered the structure is.

Detailed analysis of the experimental data (Figure 6) shows that in the XANES spectra of all samples, the features “a”, “b” and “c” appear at 347, 347.6 and 348.1 eV, respectively. These data are in good agreement with the results obtained for Ca phosphates from [61] and the positions of these spectral features are characteristic of the octahedral crystal field [61]. Moreover, in [25], it is shown that the features “a”, “b”, “c” and “e” are more prominent in the spectra of natural enamel, which is associated with higher crystallinity compared to natural dentine. It can be observed (Table 4) that “a”, “b” and “c” are shifted towards lower energies in the C_D_ biocomposite.

**Figure 7:**
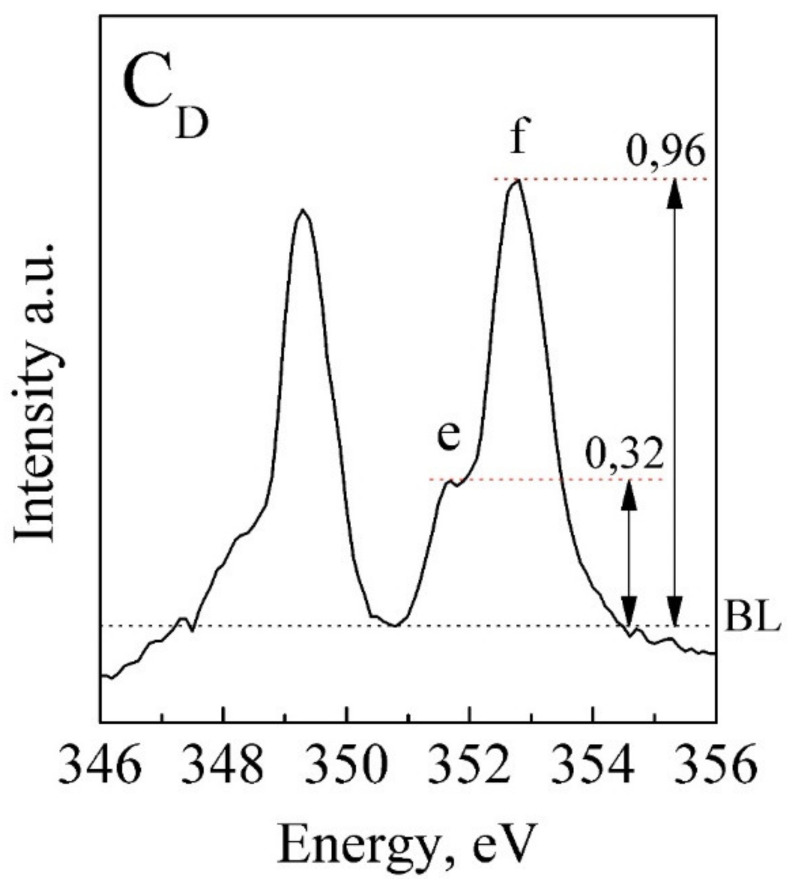
Relative heights of pre-peak “e” and peak “f” in the calcium L-edge XANES spectrum of the biocomposite C_D_. BL—baseline.

In [64], the calcium L-edge XANES spectra of calcite and aragonite were investigated. It was shown that in aragonite, which has lower symmetry than calcite, Ca is bound to nine oxygen atoms and “a”, “b” and “c” have the positions 347, 347.6 and 348.4 eV, respectively. At the same time, in calcite with higher symmetry, the Ca is surrounded by six oxygen atoms, and the positions of “a”, “b” and “c” correspond to the values 346.8, 347.2 and 348 eV, which correspond to the results obtained for the biomimetic biocomposite C_D_ (Table 4). In doing so, it was shown [63] that, for calcite, the ratio of intensities for the features “е”/“f” ~ 0.45, whereas for the C_D_ biocomposite, this ratio is ~0.3 (Figure 7), which we associated with a different Ca/P ratio.

As was shown earlier by Cosmidis et al. [61], the atomic Ca/P ratio, the fingerprint of Ca-phosphate biological tissues and biomimetic materials, can be determined using XANES data. For the calibration regression model of the Ca/P ratio, we used four standards (H_S_, H_1_, H_2_, H_3_), for which the Ca/P ratio had already been determined by the X-ray fluorescence microanalysis method (Table 2).

For each standard, we used background-corrected P and Ca L_2,3_ XANES spectra. According to the Beer–Lambert law, the step height (ΔP) in the XANES spectra of the P L_2,3_ edge measured at the energy of 144 eV is proportional to the amount of P in the volume studied. Similarly, the height (ΔCa) at 360 eV in the Ca XANES spectra is proportional to the amount of Ca in the same investigated volume. Thus, the parameter R_Ca/P_, defined as ΔCa/ΔP, is proportional to the atomic ratio Ca/P.

**Figure 8:**
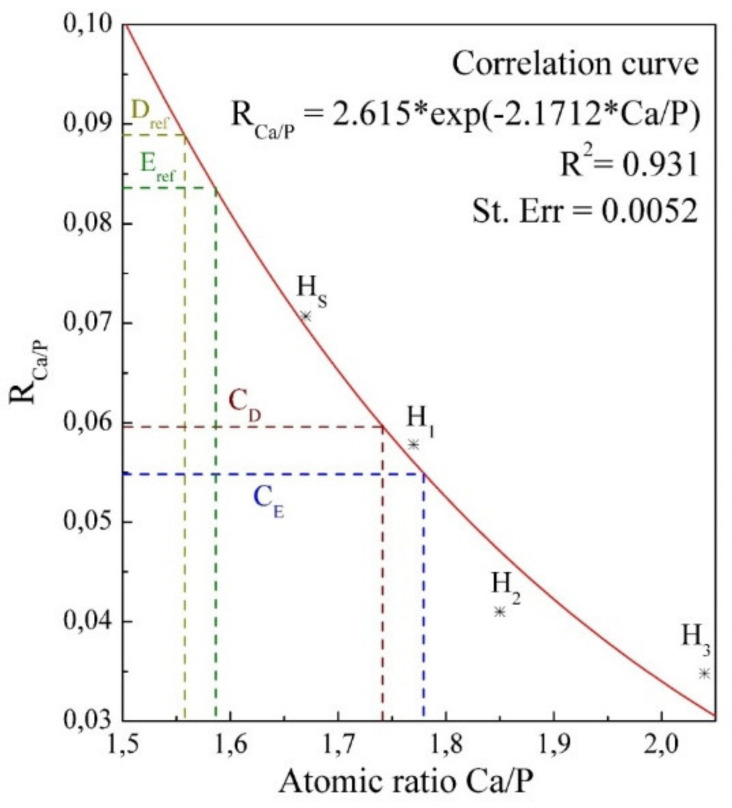
Dependence of the atomic Ca/P ratio on the experimental parameter R_Ca/P_ calculated from the XANES spectra, as well as the obtained regression curve.

Using SigmaPlot software, the correlation between the Ca/P ratio and the R_Ca/P_ parameter for the reference samples was established, and a regression curve was fitted (Figure 8). The correlation coefficient R^2^ was ~0.93. The standard error of the regression was 0.0052. In contrast to Cosmidis et al. [61], where a linear relationship was observed between Ca/P and R_Ca/P_, the regression curve we obtained has an exponential form, possibly due to the large number of standards we used.

Using the resulting regression relationship, an explicit form of which is shown in Figure 8, we determined the atomic Ca/P ratios for biological tissue samples and biomimetic composites. The calculated data are shown in Figure 8. The Ca/P ratios in our synthesised biomimetic composites, which mimic the properties of enamel and dentine, are higher than in the natural enamel and dentine from the XANES spectra of the standards [25]. However, the Ca/P relationship has a higher value in a composite that simulates the properties of enamel compared to a composite that simulates the properties of dentine. This relationship is similar to that of natural enamel and dentine, with an absolute value of:

$$\Delta {\left[{\left(\frac{Ca}{P}\right)}_{Enamel}-{\left(\frac{Ca}{P}\right)}_{Dentine}\right]}_{biomimetic}\approx \;\Delta {\left[{\left(\frac{Ca}{P}\right)}_{Enamel}-{\left(\frac{Ca}{P}\right)}_{Dentine}\right]}_{nature}$$

## 4. Discussion

Raman microspectroscopy results show that the molecular properties of the biomimetic composites that we have created are mainly inherited from the nano-cHAp and amino acid cocktail used to obtain them and are due to a given ratio between the mineral and organic components, similar to the composition of natural enamel and dentine (Figure 2, Figure 3 and Figure 4). These bioinspired composites quite satisfactorily mimic the molecular spectra of the natural mineralised tissues of enamel and dentine. The observed spectral differences in the mineral component are related to defects in the apatite structure (inclusion of various groups and ions in the nano-cHAp lattice), micro-impurities in the apatite composition, and phases of amorphous and acidic phosphate in the biogenic apatite that is present in natural tissue [32,40,43,45,51].

Thus, in the spectra of the natural samples, the vibration frequency (959 cm^−1^) and the FWHM value for the main υ_1_ PO_4_ band (Figure 3, right) are determined by the influence of acidic phosphate phases present in the natural mineralised tissue, which agrees with the available data [33,43,46,47,48,51]. At the same time, in the spectra of the bioinspired composites (C_E_ and C_D_), the position of the υ_1_ PO_4_ mode (961.8 cm^−1^) is close to the position of the same vibration in the spectrum of the stoichiometric sample (961.5 cm^−1^), and a small shift is caused by the inclusion of carbonate anions in the nano-cHAp structure used for synthesising the biocomposites. Nano-cHAp obtained by liquid-phase synthesis has B-type structural substitution (carbonate anion СO_3_^2−^ included in the position of the PO_4_ group). With the increase in CO_3_ content in the apatite structure, the υ_3_ PO_4_ mode at 1076 cm^−1^ shifts towards lower frequencies and overlaps with the υ_1_ CO_3_ vibration at 1070 cm^−1^ [32,33,46,53]. For biomimetic composites, this shift is more pronounced. At the same time, the presence of A-type substitution in the structure of natural tissues results in not only the appearance of the corresponding υ_1_ CO_3_ A-type vibration (when a carbonate anion appears in the OH position of the apatite crystal lattice) at 1106 cm^−1^ but also in the decrease in intensity of the OH group vibration at 3570 cm^−1^ (Figure 4).

The simultaneous influence of the apatite crystal size (size factor) and the degree of carbonisation on the spectral features of the mineralised tissues is important. The resulting violation of stoichiometry and the appearance of structural defects in the crystal lattice of nano-cHAp is represented in the vibrational characteristics associated with the coordination environment and the associated lattice vibrations of the phosphate ion and calcium. We used the 160–350 cm^−1^ range to consider the latter. Analysis of a set of modes located in the lattice vibration region (160–340 cm^−1^) for all the samples revealed changes in the local molecular environment of the Ca_II_ atoms for all samples. It can be seen from Figure 4 (left) that the modes related to the (Ca_II_)_3_-OH vibrations and active in the ranges of 270–290 and 320–340 cm^−1^ change their integral intensity depending on the sample type (reference, mineralised natural tissue or biomimetic composite). Thus, for synthesised nano-cHAp reference samples (H_1_, H_2_, H_3_), this phenomenon is related to the charge compensation mechanisms in the lattice and the inclusion of CO_3_ in the PO_4_ position. This leads to a decrease in the share of structurally included OH groups in the crystal lattice and is also confirmed by the change in intensity and FWHM of the OH valence vibrations in the region of 3570 cm^−1^ (Figure 4, right).

In contrast, in the 160–350 cm^−1^ region of the spectra of natural enamel and dentine, almost no modalities including (Ca_II_)_3_-OH lattice vibrations are observed, which is associated with the inclusion of the carbonate anion CO_3_ in the OH position (A-type) [57]. For the dentine specimens, this is also due to the amorphous structure and the presence of acidic phosphate phases [45,52,66]. From the point of view of lattice vibrations, for all composites (natural and synthesized), the 284, 261 and 234 cm^−1^ [41] modes of Ca-PO_4_ lattice vibrations that were observed do not change their position (Figure 4).

The difference in the intensity of these vibrations in the spectra of the samples of the biomimetic and natural composites is due to the different content and composition of the organic component, which, as shown in [40], strongly affects the vibrational density of states (VDOS). At the same time, the XANES data allowed us to identify characteristic subtle features related to the chemical environment of calcium and phosphorus in the natural mineralised tissues and biomimetic organomineral composites.

In accordance with previous work studying the local atomic environment of hydroxyapatite, fluorapatite, chlorapatite, natural enamel apatite and dentine, it has repeatedly been shown that the structure of the L_3_-edge absorption of the phosphorus atom slightly changes depending on the type of structural defect of apatite, as well as the type of isomorphic substitutions in the lattice [25,59,61,67]. In [59,68,69], it was found that for most phosphates with four oxygen atoms in the coordination environment of the phosphorus atoms, the scattering is determined by this bond, and the known isomorphous substitution does not lead to a distortion of the L_3_-edge of phosphorus. Therefore, the spectra of the biogenic phosphates and various synthetic materials have very similar spectra [26,27,61], as shown in Figure 5.

A different situation is related to the presence of a fine structure in the local environment of the calcium atoms. In the structure of apatite $Ca_{4}^{I}Ca_{6}^{\mathrm{II}}{\left(PO_{4}\right)}_{6-x}{\left(CO_{3}\right)}_{x}{\left(OH\right)}_{2-y}$, for calcium atoms, there are two different positions, which, as shown by Zougrou et al. [29], have different fine structures of the L_2,3_ edge due to different symmetries. The difference in atomic groupings in the calcium coordination environment results in both the L_3_ and L_2_ edge band broadening and the appearance of satellites in the edge region [26,29,70]. Since the crystal lattice of hydroxyapatite is composed of ~60% Ca_II_–OH group bonds and ~40% Ca^I^-PO_4_-group bonds, the greater influence of Ca^II^ on the fine structure of L_2,3_ edge satellites is a natural consequence. However, following the experimental data, the fine structure of the calcium atom edge can vary depending on the type of isomorphic substitution, the presence of vacancies in the crystal structure, and the preferential environment in HAp [64,70,71,72,73,74]. Thus, for stoichiometric HAp (H_S_), wide L_2_ and L_3_ absorption edge bands with high-intensity maxima in the 345–350 eV and 352–354 eV regions are observed in the calcium spectra. This fact correlates with known information about the dominance of the Ca^II^ state and the influence of the OH environment on the experimental width of the L_2_ and L_3_ main absorption maxima [68,75]. In turn, for the reference nano-cHAp samples (H_1_, H_2_, H_3_) synthesised using the chemical deposition method, a broadening of the L_2_ and L_3_ absorption bands is observed, but with pronounced satellites corresponding to the predominance of Ca^II^ states. Compared to the stoichiometric sample, the intensity of the satellites is reduced. This effect seems to be related to the violation of stoichiometry and the charge compensation mechanisms that lead to the exclusion of OH groups from the HAp structure [76,77].

Attention should be drawn to the type of Ca L_2_ and L_3_ absorption margins for samples of natural tooth enamel and dentine, whose shape agrees with the known data of Srot et al. [25] and is shown in Figure 6. The results of our analysis show that, for dentine specimens, and to a greater extent for enamel, the contribution of states characteristic to the environment of Ca^I^ and associated with the formation of bonds with PO_4_ tetrahedrons is significant. Concurrently, the contribution of Ca^II^ states to the spectral shape is less than for stoichiometric (H_S_) and non-stoichiometric (reference) nano-cHAp samples.

While Raman spectroscopy gives an integral picture of the molecular properties of the materials studied, it has an effective depth of ~500 nm [78], but the analysis depth of XANES spectroscopy is of ~5 nm. Considering that the crystal size of nano-cHAp in natural and inspired materials varies between 25 and 500 nm, the change in coordination environment for calcium atoms detected by XANES is typical for the surface layers of apatite nanocrystals.

To summarise our results, biomimetic organomineral composites created to reproduce physicochemical properties show a good imitation of molecular and electron energetic properties, including the carbonate anion CO_3_^2−^ and the atomic Ca/P ratio in nanocrystals. The technological operations (treatment) performed at the stage of biomimetic composite creation and their integration with natural enamel and dentine do not lead to the chemical transformations of the nano-cHAp used. Thus, the differences observed in the molecular and electron energetic structures of the natural enamel and dentine and the imitation of their properties by biomimetic materials are caused by rearrangement in the local environment of the calcium atoms in the HAp lattice.

Natural dental hard tissue formation occurs at the expense of temporary non-apatite mineral phases (ACP and OCP) and their transformation into apatite [44,45]. For this reason, the use of nanosized carbonate-substituted hydroxyapatite to create bioinspired composites and understand their features and mechanisms of formation are of great interest for a deeper understanding of the questions of HAp interaction with the organic matrix of mineralised tissues.

## 5. Limitations

This study has certain limitations connected with the spatial resolution of the diagnostics methods (XANES and Raman spectroscopy), applied for the analysis of the features related to the molecular and energetic structure of biomimetic composites and biological tissues.

## 6. Conclusions

In this work, for the first time, the influence of the coordination environment as well as the Ca and P atomic states on biomimetic composites integrated with dental tissue was investigated. Bioinspired dental composites were synthesised based on nanocrystalline calcium carbonate-substituted hydroxyapatite obtained from a biogenic source and a set of polar amino acids that modelled the organic matrix. It was shown that biomimetic composites created to reproduce the physicochemical properties of dental tissue demonstrate a good imitation of molecular and electron energetic properties, including the carbonate anion CO_3_^2−^ and the atomic Ca/P ratio in nanocrystals. The features of molecular structure of the biomimetic composites are inherited from the nano-cHAp (to a greater extent) and amino acid cocktail used for their creation and are caused by the ratio between the mineral and organic components, which is similar to the composition of natural enamel and dentine. In this case, violation in the stoichiometry of nano-cHAp, which is the mineral basis of the natural and bioinspired composites, as well as the inclusion of different molecular groups in the nano-cHAp lattice, do not affect the coordination environment of phosphorus atoms. The differences observed in the molecular and electron energetic structures of the natural enamel and dentine and the imitation of their properties by biomimetic materials are caused by rearrangement in the local environment of the calcium atoms in the HAp crystal lattice. The surface of the nano-CHAp crystals in the natural enamel and dentine involved in the formation of bonds with the organic matrix is characterised by the coordination environment of the calcium atom, corresponding to its location in the Ca^I^ position—that is, bound through the common oxygen atoms with PO_4_ tetrahedrons. At the same time, on the surface of nano-cHAp crystals in bioinspired dental materials, the calcium atom is characteristically located in the Ca^II^ position, bound to the hydroxyl OH group. The features detected in the atomic and molecular coordination environment in nano-cHAp play a fundamental role in recreating a biomimetic dental composite of the natural organomineral interaction in mineralised tissue and will help to find an optimal way to integrate the dental biocomposite with natural tissue.

## Figures and Tables

**Table 1:** Sample specifications.

Designation	Description
**H_S_**	Stoichiometric hydroxyapatite Ca_5_(PO_4_)_3_(OH)_2_ Ca/P~1.67
**H_1_**	Carbonate-substituted hydroxyapatite $Ca_{4}^{I}Ca_{6}^{\mathrm{II}}{\left(PO_{4}\right)}_{6-x}{\left(CO_{3}\right)}_{x+y}{\left(OH\right)}_{2-y}$, Ca/P~1.77 ratio
**H_2_**	Carbonate-substituted hydroxyapatite $Ca_{4}^{I}Ca_{6}^{\mathrm{II}}{\left(PO_{4}\right)}_{6-x}{\left(CO_{3}\right)}_{x+y}{\left(OH\right)}_{2-y}$, Ca/P~1.85 ratio
**H_3_**	Carbonate-substituted hydroxyapatite $Ca_{4}^{I}Ca_{6}^{\mathrm{II}}{\left(PO_{4}\right)}_{6-x}{\left(CO_{3}\right)}_{x+y}{\left(OH\right)}_{2-y}$, Ca/P~2.04 ratio
**C_E_**	A synthesised biomimetic composite that imitates the properties of tooth enamel, with 5% organic component and 95% carbonate-substituted hydroxyapatite
**C_D_**	A synthesised biomimetic composite that imitates the properties of dental dentine with 25% organic component and 75% carbonate-substituted hydroxyapatite
**E_exp_**	Natural tooth enamel
**D_exp_**	The natural dentine of the tooth
**E_ref_**	Tooth enamel, according to [25]
**D_ref_**	The dentine of the tooth, according to [25]

**Table 2:** Observed vibrations in the Raman scattering spectra of the samples.

Bond	Wavenumber, cm^−1^	Assignment	References
**Са_II_–OH**	183	Lattice	[39]
**Са–PO_4_**	204	Lattice	[39,40,41]
**Са–PO_4_**	233	Lattice	[39,40,41]
**Са–PO_4_**	265	Lattice	[39,40,41]
**Са_II_–OH**	276	Translation	[39,40,41]
**Са–PO_4_**	285–289	Libration	[39,40,41]
**Са_II_–OH**	305–312	Translation	[39,40,41,42]
**Са_II_–OH**	330	Translation	[40,42]
**υ_2_ HPO_4_**	413	OCP, dentine	[43,44,45]
**υ_2_ PO_4_**	431	O-P-O bending, υ_2_	[40,43,46,47,48,49]
**υ_2_ PO_4_**	447	O-P-O bending, υ_2_	[40,43,46,47,48,49]
**υ_4_ PO_4_**	579	O-P-O bending, υ_4_	[40,43,46,47,48,49]
**υ_4_ PO_4_**	590	O-P-O bending, υ_4_	[40,43,46,47,48,49]
**υ_4_ PO_4_**	607	O-P-O bending, υ_4_	[40,43,46,47,48,49]
**υ_4_ PO_4_**	614	O-P-O bending, υ_4_	[40,43,46,47,48,49]
**δ(CCH)**	854–857	Proline, collagen (Pro, Tyr)	[40,50]
**ν(CC)**	875	aromatic (Hyp, Tyr)	[40,50]
**υ_1_ PO_4_**	945–925	OCP, dentine	[43,44,45,51]
**υ_1_ PO_4_**	959	Enamel, dentine	[40,50]
**υ_1_ PO_4_**	962	P-O stretching	[40,43,46,47,48,49]
**ν (CC)**	1003.5	phenyl ring, dentine	[50,52]
**HPO_4_**	1005	OCP Sym stretching	[40,50]
**υ_3_ PO_4_**	1028	P-O asymmetric stretching	[32,33,40,46,53,54]
**Pyridine ring**	1034.2	Pyridine ring, dentine	[32,33,40,46,53,54]
**υ_3_ PO_4_**	1040	P-O asymmetric stretching	[32,33,40,46,53,54]
**υ_3_ PO_4_**	1047	P-O asymmetric stretching	[32,33,40,46,53,54]
**υ_3_ PO_4_**	1052	P-O asymmetric stretching	[32,33,40,46,53,54]
**υ_1_ СO_3_ B-type**	1070–1072	PO_4_ by CO_3_ substitution	[32,33,40,46,53,54]
**υ_3_ PO_4_**	1076–1077	P-O asymmetric stretching	[32,33,40,46,53,54]
**υ_1_ СO_3_ A-type**	1106	OH by CO_3_ substitution	[32,43,46]
**δ(NH)**	1241–1245, 1268	Amide III	[33,40,55]
**δ(CH)**	1450	C-H Deformation	[33,40,55]
**(C=O)/NH**	1670 m 1668 w ν	(C=O) Stretch, Amide I	[33,40,55]
**OH**	3570	OH stretch	[33,46]

**Table 3:** Position of features in the XANES spectra of the P L_2,3_-edge.

Sample	Positions of Features, eV					
	a	b	c	d	e	f
**H_S_**	136.7	137.7	138.5	139.1	141.8	147.5
**H_1_**	136.8	137.8	138.4	139.2	141.8	147.4
**H_2_**	136.7	137.7	138.4	139.2	141.8	147.5
**H_3_**	136.8	137.8	138.6	139.2	141.4	147.6
**E_Ref_** [25]	136.8	137.8	138.5	139.2	141.9	147.3
**C_E_**	136.8	137.7	138.5	139.2	141.8	147.4
**D_Ref_** [25]	136.6	137.8	138.5	139.4	142.1	147.3
**C_D_**	136.7	137.8	138.5	139.1	141.4	147.3

**Table 4:** Positions of features in the Ca L_2,3_-edge XANES spectra.

Sample	Positions of Features, eV					
	a	b	c	d	e	f
**H_S_**	-	-	348.1	349.3	351.6	352.7
**H_1_**	-	-	348.1	349.3	351.6	352.7
**H_2_**	-	-	348.1	349.3	351.6	352.7
**H_3_**	347.0	-	348.2	349.3	351.8	352.7
**E_exp_**	347.0	347.5	348.2	349.3	351.6	352.7
**E_Ref_** [25]	347.2	-	348.4	349.1	351.8	352.7
**C_E_**	347.0	347.5	348.2	349.3	351.6	352.7
**D_exp_**	347.0	347.5	348.3	349.3	351.8	352.7
**D_Ref_** [25]	-	-	348.3	349.4	351.7	352.7
**C_D_**	346.8	347.2	348.0	349.3	351.6	352.7

## Data Availability

The data that support the findings of this study are available from the corresponding author upon reasonable request.
